# Quantifying Industry Spending on Promotional Events Using Open Payments Data

**DOI:** 10.1001/jamahealthforum.2024.1581

**Published:** 2024-06-28

**Authors:** Quinn Grundy, Fabian Held, Meghan MacIsaac, Christine M. Baugh, Eric G. Campbell, Lisa Bero

**Affiliations:** 1 Lawrence Bloomberg Faculty of Nursing, University of Toronto, Toronto, Ontario, Canada; 2 Office of the Deputy Vice-Chancellor (Education), University of Sydney, Sydney, New South Wales, Australia; 3 Center for Bioethics and Humanities, University of Colorado School of Medicine, Aurora; 4 Division of General Internal Medicine, University of Colorado School of Medicine, Aurora

## Abstract

**Importance:**

Sponsorship of promotional events for health professionals is a key facet of marketing campaigns for pharmaceuticals and medical devices; however, there appears to be limited transparency regarding the scope and scale of this spending.

**Objective:**

To develop a novel method for describing the scope and quantifying the spending by US pharmaceutical and medical companies on industry-sponsored promotional events for particular products.

**Design and Setting:**

This was a cross-sectional study using records from the Centers for Medicare & Medicaid’s Open Payments database on payments made to prescribing clinicians from January 1 to December 21, 2022.

**Main Outcomes and Measures:**

An event-centric approach was used to define sponsored events as groupings of payment records with matching variables. Events were characterized by value (coffee, lunch, dinner, or banquet) and number of attendees (small vs large). To test the method, the number of and total spending for each type of event across professional groups were calculated and used to identify the top 10 products related to dinner events. To validate the method, we extracted all event details advertised on the websites of 4 state-level nurse practitioner associations that regularly hosted industry-sponsored dinner events during 2022 and compared these with events identified in the Open Payments database.

**Results:**

A total of 1 154 806 events sponsored by pharmaceutical and medical device companies were identified for 2022. Of these, 1 151 351 (99.7%) had fewer than 20 attendees, and 922 214 (80.0%) were considered to be a lunch ($10-$30 per person). Seven companies sponsored 16 031 dinners for the top 10 products. Of the 227 sponsored in-person dinner events hosted by the 4 state-level nurse practitioner associations, 168 (74.0%) matched events constructed from the Open Payments dataset.

**Conclusions and Relevance:**

These findings indicate that an event-centric analysis of Open Payments data is a valid method to understand the scope and quantify spending by pharmaceutical and medical device companies on industry-sponsored promotional events attended by prescribers. Expanding and enforcing the reporting requirements to cover all payments to all registered health professionals would improve the accuracy of estimates of the true extent of all sponsored events and their impact on clinical practice.

## Key Points

**Question:**

Is an event-centric method of analysis for Open Payments data a valid tool for quantifying pharmaceutical and medical device industry-sponsored events for health professionals?

**Finding:**

This cross-sectional study using Open Payments data identified 1 154 806 events sponsored by pharmaceutical and medical device companies during 2022 and found that 7 companies sponsored 16 031 dinners for the top 10 products.

**Meaning:**

This finding suggests that taking an event-centric approach to analysis of publicly available Open Payments data is a valid method to quantify the scope and spending on industry-sponsored events, and illustrates the limitations of the existing mandates on financial transparency.

## Teaser

This cross-sectional study using Open Payments data describes a method to quantify spending by the pharmaceutical and medical device companies on events to promote products among prescribers.

## Introduction

Promotional campaigns for pharmaceuticals and medical devices are multifaceted, encompassing advisory boards and consultant meetings, sponsored research and publications in the medical literature, and sponsorship of events for health professionals.^1,2,3^ For example, the promotion of gabapentin included events directly sponsored by the manufacturer such as speakers’ bureaus at which paid physicians presented to peers as well as continuing medical education activities funded by the manufacturer through unrestricted educational grants to third-party medical education companies.^1^ However, there are little empirical data regarding the nature, scope, scale, and spending on sponsored events, and what is known, has largely been derived from internal industry documents made public through litigation, whistleblowers, or key informants.^4^ In Australia, from 2007 through 2015, the pharmaceutical industry’s code of conduct required member companies to publicly report all details of sponsored events for registered health professionals.^5^ Analyses of these data suggest that sponsorship of events occurs frequently and is national in scope; specifically, in Australia, the pharmaceutical industry sponsored an average of 608 events for health professionals per week and more than 40% of events included attendees from multiple health professions.^6^ Furthermore, these sponsored events nearly always included food and beverages.^6^

The lack of public transparency around the nature and extent of industry sponsorship of events for health professionals makes it difficult to assess the impact that these events have on prescribing, and ultimately, on health outcomes. Events sponsored by pharmaceutical and medical device companies are designed to increase familiarity and use of promoted products among attendees and speakers as well.^3,7,8^ Analysis of the content of industry-funded continuing medical education suggests that these events are designed to overemphasize the benefits of the promoted product, minimize its risks, and exaggerate the harms of the competitors’ products.^8^ Sponsored events are often designed to promote emerging or off-label uses of brand drugs or pipeline and newly marketed products.^8^ Sometimes these drugs have cost, efficacy, and/or safety concerns.^9,10^ The evidence also suggests that sponsored events are an effective strategy for increasing prescription of promoted products: physicians who received a single sponsored meal at an event were more likely to prescribe the promoted product.^11^ Moreover, as the number of events and the total dollar value spent per physician increased, so did the likelihood of prescribing the promoted product.^11^

An event-centric perspective may be useful to understanding and quantifying the reach of promotional campaigns in terms of attendees, across clinical specialties and professions. Lawmakers recently expanded Open Payments reporting requirements to cover all prescribing clinicians including physicians, physician assistants (PAs), and advanced practice registered nurses (APRNs), which includes nurse practitioners (NPs), clinical nurse specialists, certified registered nurse anesthetists, and certified nurse-midwives.^12^ PAs and APRNs, comprise a large and growing group of prescribers, who provide care in one-quarter of all health care visits in the US.^13^ More than 70% of the 325 000 NPs in the US primary care workforce and those in full-time practice provide an average of 21 prescriptions per day.^14^ Consequently, APRNs represent an important target for pharmaceutical industry marketing: analyses of the 2021 Open Payments data found that a similar proportion of physicians, PAs, and APRNs (approximately 35%) received payments.^15^

We sought to devise a method to construct and categorize sponsored events for particular products using Open Payments records. By linking records that were plausibly associated with the same event, we aimed to quantify and describe sponsored events for particular products and to compare the characteristics of events across APRNs, physicians, and mixed audiences. Then we applied these methods to illustrate how they might be used to study promotional campaigns by identifying the top companies and drugs or devices associated with sponsored dinner events during 2022, finding patterns in promotional strategies targeted at various professional groups. We then validated our methods and typology by matching events constructed using Open Payments data with registration details provided for all industry-sponsored events on the websites of state-level NP associations.

## Methods

This study was reviewed and approved by The University of Toronto Research Ethics Board (No. 42961); informed consent was waived because all of the data are publicly available, and the study did not involve recruitment of human participants. The study followed the Strengthening the Reporting of Observational Studies in Epidemiology (STROBE) reporting guideline.

### Design and Data Sources

This was a cross-sectional study using Open Payments data (Centers for Medicare & Medicaid) for payments made from January 1 to December 21, 2022, to characterize and quantify sponsored events. We downloaded the 2022 dataset from the Open Payments website on June 30, 2023. We included all records for NPs, clinical nurse specialists, certified registered nurse anesthetists, certified nurse-midwives, and allopathic and osteopathic physicians (hereafter, physicians). To ensure consistency in the classification of these health professionals, we linked the Open Payments data to the National Plan and Provider Enumeration System data (accessed June 30, 2023) by National Provider Identifier and the National Uniform Claim Committee; individuals with ambiguous data on their provider type were excluded.

### Event-Centric Analysis to Create an Event Typology

Previous research identified that more than 90% of pharmaceutical company−sponsored events include food and beverage,^6^ and in 2021, food and beverage accounted for 91% of the total number of industry payments to physicians and 97% to APRNs.^16^ Thus, we included only payments classified as food and beverage to reliably identify distinct sponsored events. We reasoned that food and beverage would be consumed on the same day in the same place, and thus that records for food and beverage associated with the same event would share the same date of payment and location. We also assumed that the reported value of a food and beverage payment was the total cost of the hospitality, divided by the number of attendees; therefore, we grouped payment records with the same amount (rounded to the nearest dollar).

Inferring which Open Payment records were related to the same sponsored event required analytic decisions regarding the selection and representation of variables that define an event. To understand the impact of these choices, we undertook a sensitivity analysis to explore alternative ways to group Open Payments records for food and beverage, to determine how a combination of variables, including date (specific date or within the same calendar week), amount (rounded to nearest dollar), and recipient’s state may affect the identification of sponsored events in the Open Payments dataset (eTable in Supplement 1). We chose to define a sponsored event as a cluster of 3 or more individual payment records for food and beverage (nature of payment) with the following matching Open Payments record variables^17^:

- Submitting applicable manufacturer (name)
- Product category or therapeutic area
- Name of drug or biological or device or medical supply
- Recipient state
- Total amount of payment (USD, rounded to nearest dollar)
- Date of payment (exact)

After evaluating the distribution of the data, we classified events by size (large, ≥20 attendees; small, 3 to <20) and dollar amount per person. In line with detailed pharmaceutical company-reported descriptions of industry-sponsored events,^6^ we categorized events with a per person cost of less than $10 as coffee, $10 to less than $30 as lunch, $30 to less than $150 as dinner, and $150 or higher as banquet. We conducted descriptive analyses to understand the number and total spending for all types of events (coffees, lunches, dinners, and banquets, and for small and large audiences) attended by 3 different types of covered recipients: APRNs only; physicians only; and mixed (any combination of both APRNs and physicians). To illustrate the use of this method for identifying sponsored events, we identified the top products as those with the highest number of dinners across professional groups during 2022.

### Validation

To validate our typology, we identified 4 state NP associations, selected to represent 4 geographic areas (Northwest, Midwest, Southeast, and West) and which hosted many in-person industry-sponsored events in 2022 for their NP members and provided full event details on their websites to prospective attendees. We assumed that the details for these events would be as or more specific and accurate than details in Open Payments given that the information was targeted at prospective attendees. Furthermore, given that events were advertised to members of the NP association, we assumed that attendees would be mostly NPs (ie, APRNs) and that these events were dinners according to their locations (ie, restaurants) and timing (ie, evening hours) listed on the event registration websites. We extracted details for all of the in-person industry-sponsored events being hosted from the association’s web-based event listings or event calendars including event title, date, and location; sponsor; drug or medical device, if listed; and health condition, if featured.

Using our dataset of events constructed from the Open Payments food and beverage records, we assessed how many events took place overall for each of the 4 states, by type of event, and specifically, by the number of sponsored meal events (lunches, dinners, and banquets) with APRN-only audiences. Then, for each sponsored event for APRNs hosted by the NP associations, we coded the event as a match if all information for event characteristics were consistent with 1 or more events in the Open Payments dataset, or we coded it a nonmatch if 1 or more variables differed. We furthered categorized reasons for nonmatches as: (1) sponsor not a covered entity in Open Payments; (2) product not covered by Open Payments (eg, not a drug or device or not yet on the market); (3) reported in Open Payments 1 day later; and (4) no apparent match.

## Results

### Event-Centric Analysis of Open Payments Records

We identified 4 969 423 sets of Open Payments records for food and beverage payments with unique date, recipients’ state, and payment amount. Among these sets, 1 154 806 (23.2%) contained 3 or more payment records and were therefore classified as 1 event. These events represented $137 481 620 of food and beverage payments, representing 52.1% of the total spending on food and beverages to physicians and APRNs in 2022. Other combinations of identifying variables led to as few as 91 973 distinct events while covering 98.8% of total event-related payments (eTable in Supplement 1).

Using the most conservative combination of identifying variables, we identified 1 154 806 events, 1 151 351 (99.7%) of which had fewer than 20 attendees, and 922 214 (80.0%) of which we categorized as lunch (Table 1). Although dinners made up 10.4% of the total number of events, they accounted for 39.2% of the total spend on events, although manufacturers still spent more on lunch overall ($72 847 103 [53.0%]). Physicians engaged in a much larger proportion of banquets (6695 [2.3%] of 394 339) than did APRNs or mixed audiences (banquets accounted for 0.4% of APRN and 0.3% of mixed audiences) and banquets accounted for 11.9% of event spending on physicians compared with 3% on APRN and 2.6% on mixed events.

**Table 1.** Comparison of Number of Events and Total Spending on Events for APRNs Only, Mixed Audience (Both), and Physicians Only in 2022

Event type (attendees)	Total events, No. (%)^b^				Total spending, $ (%)^b^			
	APRNs only^a^	Mixed audience^c^	Physicians only	Total	APRNs only	Mixed audience	Physicians only	Total
Coffee	5017 (12.00)	66 305 (9.23)	32 750 (8.31)	104 072 (9.01)	93 268 (1.83)	1 498 701 (1.88)	630 291 (1.19)	2 222 260 (1.62)
Large (≥20)	3	35	16	54	244	4720	2706	7670
Small (<20)	5014	66 270	32 734	104 018	93 024	1 493 981	627 585	2 214 590
Lunch	30 175 (72.19)	598 952 (83.34)	293 087 (74.32)	922 214 (79.86)	1 932 781 (37.87)	49 743 155 (62.48)	21 171 167 (40.13)	72 847 103 (52.99)
Large (≥20)	62	1861	279	2202	27 381	795 126	135 865	958 372
Small (<20)	30 113	597 091	292 808	920 012	1 905 400	48 948 029	21 035 302	71 888 731
Dinner	6422 (15.36)	51 638 (7.19)	61 807 (15.67)	119 867 (10.38)	2 924 056 (57.30)	26 343 064 (33.09)	24 668 518 (46.76)	53 935 638 (39.23)
Large (≥20)	72	716	330	1118	190 201	1 929 836	787 494	2 907 531
Small (<20)	6350	50 922	61 477	118 749	2 733 855	24 413 228	23 881 024	51 028 107
Banquet	184 (0.44)	1774 (0.25)	6695 (2.29)	8653 (0.75)	153 115 (3.00)	2 033 366 (2.55)	6 290 138 (11.92)	8 476 619 (6.17)
Large (≥20)	1	32	48	81	3864	136 952	248 548	389 364
Small (<20)	183	1742	6647	8572	149 251	1 896 414	6 041 590	8 087 255
Total	41 798 (100)	718 669 (100)	394 339 (100)	1 154 806 (100)	5 103 220 (100)	79 618 286 (100)	52 760 114 (100)	137 481 620 (100)

Abbreviation: APRNs, advanced practice registered nurses.

^a^ APRNs includes nurse practitioners, clinical nurse specialists, certified nurse-midwives, and certified registered nurse anesthetists.

^b^ All percentages are column percentages.

^c^ Mixed audiences included 2 or more professional groups (APRNs, physician assistants, and/or physicians).

To illustrate the use of this method, we selected the top 10 products based on the number of sponsored dinner events for physicians, PAs, and APRNs. Seven companies sponsored the events for the top 10 health products in terms of number of associated dinner events (Table 2). For 8 of the 10 top products, most dinner events included mixed audiences. However, for 2 products there were more APRN-only events than physician-only events, although the most dinners were still for mixed audiences: 253 events for APRNs (vs 181 for physicians) associated with valbenazine tosylate, a vesicular monoamine transporter 2 inhibitor indicated for tardive dyskinesia and chorea; and 224 APRN dinners (vs 157 for physicians) for cariprazine, an atypical antipsychotic indicated for treatment of schizophrenia and bipolar disorder and as an adjunctive therapy to antidepressants in major depression. For a surgical system, there were almost no events for audiences that did not include physicians. For the drugs, dupilumab (a monoclonal antibody for allergic diseases) and upadacitinib (indicated for moderate to severe rheumatoid and psoriatic arthritis, inflammatory bowel disease, atopic dermatitis, and ankylosing spondylitis^18^), there were more physician-only dinners than for mixed or APRN audiences.

**Table 2.** Audience Type (APRNs, Physicians, or Mixed/Both) and Spending on Events for the Top 10 Products Based on the Number of Sponsored Open Payments Dinners ($30 to <$150 per Person) in 2022

Company and product(s)	Total events, No.				Total spending, $			
	APRNs only	Mixed audience	Physicians only	Total	APRNs only	Mixed audience	Physicians only	Total
AbbVie								
Upadacitinib	49	845	972	1866	16 636	365 919	403 702	786 257
Risankizumab-rzaa	52	576	534	1162	18 903	268 033	215 236	502 172
Ubrogepant	80	927	408	1415	34 666	472 075	157 628	664 369
Cariprazine	224	1131	157	1512	116 091	721 878	64 401	902 370
Bayer HealthCare Pharmaceuticals								
Finerenone	49	958	562	1569	26 902	523 170	222 279	772 351
Boehringer Ingelheim Pharmaceuticals								
Empagliflozin	31	823	421	1275	11 876	501 321	183 399	696 596
Intuitive surgical								
Robotic surgical system	4	155	1309	1468	539	91 375	509 232	601 146
Janssen Pharmaceuticals								
Rivaroxaban	98	1061	998	2157	38 781	575 534	447 029	1 061 344
Neurocrine BioSciences								
Valbenazine tosylate	253	711	181	1145	115 944	374 102	64 434	554 480
Sanofi Genzyme/Regeneron Pharmaceuticals								
Dupilumab	37	1109	1316	2462	12 990	322 676	411 602	747 268

Abbreviation: APRNs, advanced practice registered nurses.

### Validation

We identified 131 200 meal events across the 4 states, 23 050 dinners, and 776 dinners specifically for APRNs only (Table 3). The sponsored events hosted by the 4 NP associations represented 0.2% (227 of 131 200) of all meal events; 1.0% (227 of 23 050) of dinners; and 29.3% (227 of 776) of APRN-only dinners. The registration details for 168 of the 227 meals (74.0%) matched events constructed from Open Payments data; however, we had misclassified 10 dinners as lunch (ie, these dinners cost <$30 per person) and for 22, the otherwise matching Open Payments records did not specify an associated product or therapeutic area. For 19 events (8.4%), the registration details matched more than 1 and as many as 6 Open Payments events, suggesting sponsors may have hosted multiple events for the same product on the same date. Among those that had no matching Open Payments event (59 of 227 [26.0%]), 12 (5.3%) matched an Open Payments event reported for the previous or the next day, and 19 (8.4%) were sponsored by companies that were not covered entities or for products that were not covered by Medicare (eg, diagnostic or genetic tests). Table 4 provides illustrative examples of industry-sponsored events hosted by the NP associations that matched or did not match Open Payments events.

**Table 3.** Open Payments Events Typology Validated Against Sponsored Dinner Events Hosted by 4 State-Level NP Associations in 2022

Method	Variable, No.	California	Kentucky^a^	Massachusetts	Michigan	Total
Open-payments data	Meal events Lunches Dinners/banquets	84 297 67 913 16 384	8525 7850 675	9244 7296 1948	29 134 25 091 4043	131 200 108 150 23 050
	APRN-only meal events Lunches Dinners/banquets	1178 792 386	627 567 60	392 248 144	825 639 186	3022 2246 776
State NP associations	Total events	114	22	44	47	227
	Match^b^ (% of total events) Product NS^c^ Lunch^d^	80 (70) 17 5	15 (68.1) 2 0	35 (79.5) 3 1	38 (80.9) 0 4	168 (74.0) 22 10
	Nonmatch (% of total events) Not covered^e^ ±1 Day No match	34 (29.8) 10 9 15	7 (31.8) 4 1 2	9 (20.5) 6 2 1	9 (19.1) 4 1 4	59 (26.0) 19 12 28

Abbreviations: APRN, advanced practice registered nurse; NP, nurse practitioner; NS, not specified.

^a^ Kentucky NP association reported events only for September to December 2022, so validation with Open Payments dataset was restricted to September 1 to December 31, 2022, for Kentucky.

^b^ Match was defined as dinner events for APRN-only or mixed audiences with same date, sponsor, state, and product and/or therapeutic area.

^c^ All event characteristics match, but associated product and/or therapeutic area was not specified in Open Payments dataset.

^d^ All event characteristics match, but identified as lunch instead of dinner in Open Payments dataset.

^e^ Either the sponsor (ie, applicable manufacturer making payment) or product was not a covered entity under the Open Payments reporting requirements.

**Table 4.** Illustrative Industry-Sponsored Dinner Events Hosted by 4 State-Level NP Associations in 2022

Open Payments match	Event date	Event title, city, state	Location	Sponsor	Speaker credentials	Details^a^
No (not a covered product)	November 3, 2022	Genetics in Clinical Practice; Ironton, Kentucky	Restaurant	Myriad	PhD, PMHNP-BC, APRN, MSN	Target audience: NPs Learning goal: discussing genetics in clinical practice Learning objectives: genetics in clinical practice and clinical decision-making Genesite instruction method: lecture Maximum registrants: 40
No	June 7, 2022	Advanced Wound Care Portfolio; Kalamazoo, Michigan	Restaurant	Smith+ Nephew	RN, BSN	A preventable problem. A comprehensive solution. Together we can help reduce preventable pressure injuries and improve outcomes. Products. Practice. Protocols.
No (not a covered entity)	November 17, 2022	Unraveling ALS: A Closer Look at the Importance of Early Diagnosis and the Underlying Complex Pathophysiology; Irvine, California	Restaurant	Amylyx	MD	Free bonus OC Chapter November education and business meeting 2022
No	September 21, 2022	Advances in the Treatment of Insomnia; Newton, Massachusetts	Restaurant	Idorsia	MD	Join us for dinner, networking, and a great educational program on: Advances in the Treatment of Insomnia
No (not a covered entity)	August 11, 2022	An Innovation in Gentle Formula: Closer to Human Milk Proteins Drive Clinical Benefits; Anaheim, California	Restaurant	By Heart	MD	OC Chapter August Education and Business Meeting 2022
Yes, matched 1 dinner event but NS product	October 12, 2022	Understanding the emotional and physical burdens of patients with hidradenitis suppurativa (HS)^b^; Louisville, Kentucky	Restaurant	AbbVie	MD	Target audience: nurse practitioners Learning goal: discuss hidradenitis suppurativa and its effect on emotional and physical burden. Instruction method: live speaker with presentation
Yes (3 events)	September 21, 2022	Distinguishing Unipolar From Bipolar Depression; Santa Rosa, California	Restaurant	AbbVie	NP	September dinner meeting, A Clinical Overview of VRAYLAR (cariprazine): Making the Diagnosis 6:00-6:15 arrival 6:15 business meeting 6:30 dinner and presentation
Yes (exact match)	August 29, 2022	Tailoring a Postmenopausal Osteoporosis Management Plan Specific to Your Patient; Grand Rapids, Michigan	Restaurant	Amgen	MD, FACP, FACR	Your chance to hear from an experienced peer
Yes (exact match)	September 22, 2022	Redefining Treatment of X-Linked Hypophosphatemia; Prestonsburg, Kentucky	Restaurant	Ultragenyx	MSN RN	Target audience: nurse practitioners Learning goal: identification and treatment of low phosphorus in patients (6 mo-adult). Learning objectives: overview of role of phosphorus and assessment in primary care of patients with decreased phosphorus. Instruction method: lecture Director’s comments: this program will provide wealth of information!
Yes (exact match)	June 16, 2022	SPRAVATO and the Management of Two Subtypes of Challenging to Treat Major Depressive Disorder in Adults; Worcester, Massachusetts	Restaurant	Janssen Pharmaceuticals	PA-C	Join us for dinner, networking, and a great educational program on: The Management of Two Subtypes of Challenging to Treat Major Depressive Disorder in Adults

Abbreviations: APRNs, advanced practice registered nurse; BSN, Bachelor of Science in Nursing; FACP, fellow of the American College of Physicians; FACR, fellow of the American College of Radiology; MSN, Master of Science in Nursing; NP, nurse practitioner; NS, not specified; PA-C, physician assistant, certified; PhD, doctor of philosophy; PMHNP-BC, psychiatric mental health nurse practitioner-board certified; RN, registered nurse.

^a^ Copied verbatim from the event’s internet registration page.

^b^ Upadacitinib, marketed by AbbVie, is currently under clinical trial for this indication.^18^

The events hosted by the 4 state-level NP associations also reflect a facet of larger promotional campaigns for the top 10 products based on the highest number of sponsored dinners in Open Payments. Of the 227 events hosted by the NP associations, 47 (20.7%) were sponsored by manufacturers of the top 10 products, including 35 events (15.4%) explicitly associated with 1 of the top 10 products and approved indications, or in 1 case, an indication currently under clinical trial.

## Discussion

Our event-centric analysis of Open Payments data suggests that pharmaceutical and medical device companies are sponsoring a minimum of 3000 events a day for prescribing clinicians, suggesting that sponsored events are part of their national promotional campaigns targeted across professional groups. An event-centric analysis is a valid method to understand the nature and quantify the scope of manufacturer-sponsored events in the context of a product’s promotional campaign. However, the true extent of the scope of all sponsored events targeted at prescribing clinicians is likely an underestimate given that our parameters for defining an event were conservative and because certain manufacturers and products are exempt from reporting requirements. Previous Open Payments analyses have largely taken an individual and grouped prescriber-centric approach, finding that receipt of payments is associated with increased prescribing quantity and cost^19^ and that the likelihood of receiving a payment increases when those within a physician’s professional referral network also receive payments.^20,21,22^ An event-centric approach allows for a better understanding of the nature and impacts of prescriber-industry interactions in the social contexts in which they occur.

Future research could explore the impact of drug promotion on a professional group or clinical specialty, and the ways that promotion may variably target different groups. Detailed analyses of events for highly promoted drugs could also shed light on the breadth of industry promotional strategies, for example, by comparing the prevalence of events vs individual payments for consulting or advisory board membership associated with different products. Analyses of multiple years of data could also assess the timing of sponsored events in relation to key points in a drug’s life cycle (eg, market access, Medicare reimbursement, and patent status).

The findings of this analysis suggest that there is a need for key policy actions that further expand and enforce Open Payments reporting requirements to achieve transparency goals. Among a sample of industry-sponsored dinner events hosted by state-level NP associations, we were only able to match approximately three-quarters of events to those in Open Payments. Many of the missing events were sponsored by manufacturers whose products were not covered by Medicare, such as genetic or diagnostic tests or products not yet on the market, and thus, were not eligible for inclusion in the Open Payments database.

Given the proven impacts on prescribing outcomes, commercial entities—regardless of their industry or product portfolio—should report the provision of payments, meals, and gifts to any registered health professional, including trainees, such as medical residents.^23^ Because dinner events were publicly advertised to prospective attendees through professional associations, it is likely that more informal coffee- and lunch-type events may be even more infrequently reported. However, previous analyses suggest that most sponsored events occur in clinical settings,^6^ which suggests that health care facilities should have a greater role in ensuring transparency and/or regulating industry-sponsored events.

### Limitations

This study had some limitations. We underestimated the total number of sponsored events, both within a promotional campaign and overall. First, the method may not apply to virtual sponsored events. We noted a small number of sponsored virtual events hosted by the state-level NP associations during 2022— a decrease from the previous year when most events were online due to the COVID-19 pandemic and offered attendees a gift card to a food delivery company. However, because we could not determine the relationship between the date of the event and the date that the payment was reported, we excluded virtual events from this analysis. Second, we grouped payment records to recipients whose primary practice address was in the same state. Thus, we missed events for which recipients traveled out of state to events such as national or regional conferences. However, the use of restrictive matching criteria may have also minimized identifying false positives, such as meals provided through consulting engagements involving recipients from multiple states. Although we validated our method using complete and accurate sources of data on industry-sponsored events occurring within a single state and advertised to NPs, we identified few other publicly available data sources that provided the level of detail that would enable validation of other Open Payments events. Thus, we cannot know the proportion of identified events that did not represent an actual event.

## Conclusions

The findings of this cross-sectional study suggest that taking an event-centric approach to analysis of publicly available Open Payments data is a valid method for quantifying the scope and spending of pharmaceutical and medical device companies on promotional events attended by APRNs and physicians. These findings also uncover the limitations of the existing mandates on true financial transparency. Open Payments data could allow for event-centric analyses to better understand the extent to which sponsored education and other activities are part of multifaceted, systemic, and strategic promotional campaigns and to assess their impact on prescribing. Efforts to expand and enforce Open Payments mandates to include payments and meals from commercial entities to all registered health professionals, regardless of the product promoted, would further advance transparency’s goals.

## Notes

Supplement 1. eTable. Sensitivity analysis of alternative ways to group events

Supplement 2. Data Sharing Statement
