# Supplementary material for: Quantifying Industry Spending on Promotional Events Using Open Payments Data
Source: JAMA Health Forum. 2024 Jun 28;5(6):e241581. doi: 10.1001/jamahealthforum.2024.1581 (PMC11214116; doi:10.1001/jamahealthforum.2024.1581)
Supplement: Supplement 1. — eTable. Sensitivity analysis of alternative ways to group events [file jamahealthforum-e241581-s001.pdf]

Supplemental Online Content

Grundy Q, Held F, MacIsaac M, Baugh CM, Campbell EG, Bero L. Quantifying Industry Spending on Promotional Events Using Open Payments Data. *JAMA Health Forum*. 2024;5(6):e241581. doi:10.1001/jamahealthforum.2024.1581

eTable. Sensitivity analysis of alternative ways to group events

This supplemental material has been provided by the authors to give readers additional information about their work.

© 2024 Grundy et al. *JAMA Health Forum.*

eTable. Sensitivity analysis of alternative ways to group events

Variables to identify sets of distinct payments	Distinct sets of payment entries	Sets of payment entries with three or more associated recipient IDs, "events"	Sets of large events with twenty or more associated recipient IDs	Mean attendees for events	Median attendees for events	Sum of payments attributed to events (USD)	Events with recipients from a single state
Date	417,243	256,643 (61.5%)	84,087 (32.8%)	37	10	254,277,970 (96.3%)	21,931 (15.0%)
Date, amount	2,331,180	979,843 (42%)	77,940 (8%)	8	5	206,758,976 (78.3%)	231,967 (34.9%)
Date, recipient state	2,375,866	965,918 (40.7%)	68,667 (7.1%)	8	5	199,855,060 (75.7%)	965,918 (100%)
Date, recipient state, amount	4,969,423	1,154,806 (23.2%)	3,455 (0.3%)	4	4	137,481,620 (52.1%)	1,154,806 (100%)
Week	137,021	91,973 (67.1%)	41,067 (44.7%)	101	15	260,791,334 (98.8%)	5,771 (11.4%)
Week, amount	1,270,230	612,895 (48.3%)	104,478 (17%)	15	6	224,501,706 (85%)	104,016 (26.4%)
Week, recipient state	1,132,965	581,509 (51.3%)	103,177 (17.7%)	15	7	230,993,350 (87.5%)	581,509 (100%)
Week, recipient state, amount	3,912,922	1,176,790 (30.1%)	17,952 (1.5%)	6	4	160,031,910 (60.6%)	1,176,790 (100%)

© 2024 Grundy et al. *JAMA Health Forum.*
