# Supplementary material for: Quantifying Industry Spending on Promotional Events Using Open Payments Data
Source: JAMA Health Forum. 2024 Jun 28;5(6):e241581. doi: 10.1001/jamahealthforum.2024.1581 (PMC11214116; doi:10.1001/jamahealthforum.2024.1581)
Supplement: Supplement 2. — Data Sharing Statement [file jamahealthforum-e241581-s002.pdf]

Data Sharing Statement

Grundy. Quantifying Industry Spending on Promotional Events Using Open Payments Data. *JAMA Health Forum*. Published June 28, 2024. doi:10.1001/jamahealthforum.2024.1581

Data
Data available: Yes
Data types: Data (not involving human participants)
How to access data: All data pertaining to payment records are publicly available for download from the Center for Medicare and Medicaid Services Open Payments website at: https://openpaymentsdata.cms.gov and NPPES https://download.cms.gov/nppes/NPI_Files.html
When available: With publication

Supporting Documents
Document types: Statistical/analytic code
How to access documents: The statistical code used to classification sponsored events is publicly available through the Borealis Dataverse at: https://doi.org/10.5683/SP3/0KR09P.
When available: With publication

Additional Information
Who can access the data: The data are publicly available.
Types of analyses: The data are publicly available.
Mechanisms of data availability: The data are publicly available.
